# The correlates and course of multiple health risk behaviour in adolescence

**DOI:** 10.1186/s12889-016-3120-z

**Published:** 2016-05-31

**Authors:** Daniel R. Hale, Russell M. Viner

**Affiliations:** The Policy Research Unit in the Health of Children, Young People and Families, General and Adolescent Pediatrics, Institute of Child Health, UCL, 30 Guilford St., London, WC1N 1EH UK

**Keywords:** Adolescence, Health, Risk behaviours, Substance use

## Abstract

**Background:**

Health risk behaviours often co-occur in adolescence. This may be partially explained by a set of common risk and protective factors. The current study examines the association between risk behaviours throughout adolescence and identifies common risk factors for multiple risk behaviour in late adolescence.

**Methods:**

We use data from the Longitudinal Study of Young People in England. We examined the association between risk behaviours at age 14 (*n* = 15,588), age 16 (*n* = 12,416) and age 19 (*n* = 9,548). The associations between age 19 risk behaviour and earlier risk behaviours and risk and protective factors were assessed longitudinally. Health risk behaviours included smoking, alcohol use, illicit drug use, delinquency and unsafe sexual behaviour.

**Results:**

All risk behaviours were found to be associated with other risk behaviours with associations weakening through adolescence. A number of sociodemographic, interpersonal, school and family factors at age 14 predicted risk behaviour and multiple risk behaviour at 19, though predictors for heavy alcohol use often differed from other health risk behaviours. Past risk behaviour was a strong predictor of age 19 risk behaviour though many involved in only one form of risk behaviour in mid-adolescence do not progress to multiple risk behaviour.

**Conclusions:**

Our findings reaffirm the links between health risk behaviours, but these diminish throughout adolescence with multiple risk behaviour usually initiated in mid-adolescence. Multiple risk behaviour is initiated in early or mid adolescence with a number of common risk factors explaining the co-occurrence of risk behaviours.

## Background

Many behaviours linked to illness, injury, mortality or other negative outcomes have their origin in adolescence [1, 2]. Approximately 80 % of lifetime smoking and alcohol use is initiated in adolescence in both high and middle-income countries and initiation of illicit drug use is rare over age 25 years [3]. Age of initiation of sexual risk behaviours is predominantly in the teens in most countries [4]. Similarly, adolescent aggression and delinquency remain moderately stable into adulthood [5]. These risk behaviours are linked with a range of adverse outcomes in later life [5–9] which have made the prevention of adolescent health risk behaviours a policy focus both in the UK and internationally [10].

Adolescent health risk behaviours such as delinquency, substance use and sexual risk often co-occur [11–15]. Correlations between risk behaviours have typically been found between .03 and 0.6 [16, 17]. There are two broad theoretical approaches to explaining associations between health risk behaviours. Gateway theories purport that involvement in one form of risk leads to others through increased exposure and desire to engage in other unsafe behaviours or through decreases in the perceived danger of other risk behaviours [18]. There is some empirical support for these theories with smoking and alcohol use leading to exposure and increased initiation of drug use [19] and sexual risk behaviour [20, 21]. Another approach is to postulate a set of common risk factors for risk behaviours. Jessor’s “problem behaviour theory” suggests that behaviours which are socially-defined as problematic or unconventional are enacted as a manifestation of disregard for such social conventions. This proclivity for problem behaviour arises based on psychosocial protective and risk factors, with protective factors decreasing the likelihood of problem behaviour and risk factors increasing the likelihood. Because conventions for health behaviours are often age-dependent, early health-risk behaviour is often enacted as a means of demonstrating maturity and independence and repudiating conventionality. Within problem behaviour theory, this shared function across risk behaviours in adolescence is seen as a core explanation for co-occurrence [22]. The identification of a number of common risk and protective factors for risk behaviours [11, 14, 23] has contributed to an empirical basis for problem behaviour theory.

However, little is known about the course of co-occurrence throughout adolescence. This information could both help inform interventions as well as cast light on the mechanisms of co-occurrence. For instance, gateway theories would predict an ever-increasing association between risk behaviours as adolescents gained more and more opportunities to pursue new risk behaviours. Problem behaviour theory, however, might suggest that adolescent experimentation with health risk behaviour, having correlating meanings and function, might settled into stable adult health behaviour in late adolescence [24], co-occurrence might diminish. A recent study indicated that the shared etiology contributing to smoking, alcohol and marijuana use diminished through adolescence and early adulthood [25]. This suggests that interventions targeting multiple risk behaviours are particularly relevant in early adolescence.

The current study aims to:

1. Examine associations between risk behaviours throughout adolescence into early adulthood to:
  - Examine magnitude of associations across risk behaviours
  - Assess changes in the magnitude of associations across adolescence.
2. Identify common risk factors in early adolescence for alcohol use, drug use and sexual risk behaviour in later adolescence, and
3. Identify patterns of early risk behaviour associated with multiple risk behaviour in late adolescence.

We hypothesise, based on problem behaviour theory, that associations between risk behaviours will be of greater magnitude for risk behaviours which most precisely represent the aim of repudiating age-delineated social norms. That is, behaviours which are strongly socially condemned will have stronger average associations, due to their shared etiological function, than behaviours which are considered age-normative or conventional. Specifically, we predict the weakest associations with alcohol use, which is often considered normative within adolescence [26]. Conversely, we expected relatively high associations with illicit drug use due to its perceived danger and unconventionality [27]. No hypotheses regarding the magnitude of associations across adolescence are proposed due to convergent predictions from gateway theories and problem behaviour theories.

In line, with past literature, we expect to find common psychosocial risk factors across risk behaviours. However, problem behaviour theory would predict that, as with associations among risk behaviours, less conventional behaviours would have more similar patterns of risk. As such, we postulate the most divergent pattern of risk factors for adolescent alcohol use. We hypothesise that early risk behaviour, particularly multiple risk behaviour in early adolescence, will predict early adult multiple risk behaviour.

## Methods

This study used data from the Longitudinal Study of Young People in England (LSYPE) managed by the Department for Education. Detailed methodology for the panel study has been published previously [28]. Begun in 2004 when respondents were in Year 9 (an educational year group in the UK taken when students are approx. age 14), the panel was followed-up annually until 2010, resulting in a total of seven waves of data. Our analyses focus predominantly on data in the first three waves and in wave 6. The data is publically available from the UK Data Service (http://ukdataservice.ac.uk). As the study used only secondary data, ethical approval was not required, as per the UCL Research Ethics Committee guidelines.

892 schools throughout England were selected for inclusion at wave 1 with 647 (73 %) agreeing to participate resulting in a nationally-representative total of 15,588 respondents. In subsequent waves, dropout rates ranged from 14 to 8 %, with a sample of 9,548 in the sixth wave. Data were collected via face-to-face interviews with respondents and their parents. These data are supplemented by National Pupil Data (NPD) linked to LSYPE responses. The NPD is a pupil level administrative record containing information for all state-educated students in England including attainment and school history.

### Measures

All measures used in the study were self-reported by young people except for two measures of family environment (how well young person gets along with parents and whether young person lives in single parent household) which were parent reported, as were school exclusions and parent education. Academic attainment was obtained from the NPD. Area-level deprivation was determined based on respondents’ postcodes.

We used data from Waves 1 to 3 and Wave 6. We did not include data from Waves 4 and 5 because of a lack of information on risk behaviours. Wave 4 did not include items on smoking or sexual risk, making it unsuitable for comparing across a range of risk behaviours to earlier and later waves. Wave 5 did not include any assessment of risk behaviours.

#### Health risk behaviours

Health risk behaviours were chosen (based on their availability within the dataset) that represented common adolescent risks. Risk behaviours at age 14 (wave 1) and age 16 (wave 3) consisted of smoking, alcohol use, cannabis use and delinquency. We created binary risk behaviour items as per government national statistics of substance use which use the same data set [11]. Regular smoking was defined as smoking an average of at least one cigarette a week, and regular alcohol use as drinking on average at least once a week. Cannabis use was a binary measure of whether the respondent had reported ever trying cannabis. Finally, delinquency at age 14 was defined as having ever vandalized public property, shoplifted, graffitied or taken part in a fight or public disturbance. At age 16, delinquency was defined as any of the same behaviours within the last year. This change was due to the availability of items within the survey. Not all risk behaviours were available across all waves. Sexual risk was not available until wave 6. At age 19 (wave 6) heavy alcohol use was defined as drinking at least three or four times a week on average. Drug use was defined as having used any drugs in the last four weeks. Unsafe sex was defined based on two measures: whether the respondent reports ever having had unsafe safe, and, for those who report having had unprotected sex, the frequency of unsafe sex (with response options including “rarely”, “less than half the time”, “around half the time”, “most times”, or “always”. We defined unsafe sex as having ever had unprotected sex and reporting frequency from “less than half the time” to “always”. The only measures of drug taking available in wave 1 and wave 3 were having ever tried cannabis. No measures of delinquency and smoking were available in wave 6.

We calculated measures of multiple risk behaviour at each of age 14, 16 and 19. Multiple risk behaviour was defined as involvement in two or more risk behaviours (as defined above) during any one wave.

#### Postulated predictors of health risk behaviours

Predictors of age 19 behaviours were categorized into socio-demographic factors, previous risk behaviour, interpersonal factors, school, and family.

##### Socio-demographic factors

Socio-demographic factors included gender and ethnicity reported at age 14 and socio-economic status. The latter was based on 2004 English Index of Multiple Deprivation (IMD) quintiles and parental education. The IMD is an area-level measure of deprivation created by the UK’s British Department for Communities and Local Government which categorises areas in England based on seven domains of deprivation including income, employment, health, education and training, housing and services, living environment and crime. These domains are ranked and combined to create overall area-level deprivation scores [29]. Respondents self-categorized into sixteen ethnicities which, for the purpose of the current study were collapsed into five: white, south Asian, black, mixed race, and other ethnicities. Parental education was defined based on whether either parent reported having any secondary school qualifications.

##### Previous risk behaviour

Previous risk behaviour was based on reports of age 14 and age 16 risk behaviour as described above. For each of regular smoking, regular alcohol use and delinquency, a four-category variable was defined indicating those who were never involved in that risk behaviour, those involved only at age 14, those involved only at age 16 and those involved at both reporting periods. For cannabis use, respondents were categorized as having never tried it, tried it by age 14, or tried it by age 16. Finally, a ‘risk score’ was constructed for both age 14 and age 16 by summing the number of health risk behaviours the respondent reported involvement in.

##### Interpersonal measures

Psychological distress was assessed using the General Health Questionnaire (GHQ), completed at age 15. A12-item measure of recent psychological distress, it includes items regarding a personal sense of self-worth, self-confidence and enjoyment of day-to-day activities [30]. As per UK national statistics criteria, scores of 4 and above were considered indicative of psychological distress [31]. Three single-item measures reported at age 14 assessed relationships with peers. Having friends over often and going out with friends often were defined as more than twice a week. A binary measure of bullying indicated whether respondents reported being a victim of any form of bullying in the last 12 months.

##### School

Attainment was measured as the average of English, maths and science Key Stage 3 exams which are usually administered when pupils are 14 years old. Low academic attainment was defined as falling below the expected level of attainment as per the UK National Curriculum (Level 5). All other school variables were self-reported at age 14. Included was a binary self-assessed measure of academic ability, distinguishing those who report being average or worse at schoolwork from those who report being above average. Truancy was defined as any truancy in the last 12 months. School exclusion was defined as having been temporarily suspended or permanently excluded from school. Finally, attitudes towards school were assessed with a composite measure based on responses to 12 items about how worthwhile, interesting and enjoyable respondent’s felt school is.

##### Family

Family environment was assessed based on four items reported at age 14. One item indicated whether the young person lived in a single-parent household. The three others assessed the relationship with their parents. A parent-reported item assessed how well the respondent got along with parents, classified as ‘fairly or very well’ or ‘fairly or very badly’. Parental communication was measured as the extent to which the respondent talked to either of their parents about things that are important to them, classified as less than or more than once a week. Finally, respondents assessed how often parents knew where they were while out in the evening classified as ‘always’ or ‘not always’.

### Analyses

All analyses were weighted to account for unequal probabilities of selection and follow-up non-response, using weighting variables provided by the LSYPE team. Variables for which unequal probability of follow-up were weighted include region, ethnicity, academic achievement, gender, socio-economic status and risk behaviour participation. To account for the clustered sampling protocol, analyses were carried out in Stata 12 [32] using the survey estimation commands (svy); this accounts for sampling strata, primary sampling unit and design and attrition weights.

We first carried out separate logistic regression models assessing the association between each risk behaviour and other risk behaviours at each reporting period. Analyses were controlled socio-demographic variables including area-level deprivation, gender and ethnicity which have all been shown to be independently associated with health risk behaviours in adolescence. A series of logistic regression models were run to assess the association between early (age 14 and age 16) risk behaviour, family, school and interpersonal factors, and age 19 risk behaviours, controlling for area-level deprivation, gender and ethnicity. Finally, two chi-square analyses were conducted comparing the proportion of young people involved in each type of risk behaviour, multiple risk behaviour (two or more health risk behaviours) and no risk behaviours at age 14 and age 16 who, at age 19 were involved in multiple risk behaviour. Expected and observed counts were computed, as well as adjusted residuals which indicate the magnitude of the difference between observed and expected counts.

## Results

Data were available on 15,588 at age 14, of which 12,416 were retained at age 16 and 9,548 at age 19. At age 14, 66 % of the sample self-identified as white, 17 % Asian, 9 % black, 5 % mixed and 3 % ‘other’ ethnicity. 51 % of the sample was male.

### Attrition analyses

We compared rates of survey completion at age 19 based on key socio-demographic variables and risk behaviour participation at age 14. Attrition was significantly higher in males (41 %) than females (36 %; *p* < .001). Follow-up also differed based on ethnicity (*p* < .001). Black respondents had the highest rates of attrition (51 %). Mixed ethnicity respondents had an attrition rate of 44 %, followed by Asian (38 %) and white respondents (37 %). Those who did not categorise into these ethnicities (‘other’ ethnicity) had an attrition rate of 47 %. Finally, attrition rose steadily from the least deprived (22 %) to the most deprived IMD quintile (38 %; *p* < .001).

No significant differences in follow-up were found between regular alcohol users and non-regular users with attrition rates of 41 and 38 % respectively (*p* = .09). However, regular smokers had substantially higher attrition (59 %) than non-smokers (37 %; *p* < .001). Likewise, those who had tried cannabis by age 14 were more likely to drop out (46 %) than those who had not (38 %; *p* < .001). Finally, those involved in delinquent behaviour had higher attrition rates (47 %) than those reporting no involvement (35 %; *p* < .001). Overall, the analyses suggest differential attrition across socio-demographic variables and risk behaviour participation. The following analyses use sample weighting to partially attenuate this shortcoming.

### Prevalence of individual and concurrent risk behaviours

The prevalences of individual risk behaviours and combinations of behaviours at age 14 and 16 are shown by gender in Table 1, with those for age 19 in Table 2. (‘Individual’ risk behaviour use refers to involvement in a given risk behaviour without regard to involvement in other risk behaviours, i.e., involvement in an individual risk behaviour may or may not co-occur with other risk behaviours).

**Table 1:** Prevalence of risk behaviours by gender at age 14 and age 16

	Age 14		Age 16	
	Male	Female	Male	Female
	*N* = 7,939	*N* = 7,649	*N* = 6.286	*N* = 6,130
	% (95 % CI)	% (95 % CI)	% (95 % CI)	% (95 % CI)
Regular smoking	4.5 (3.8, 5.2)	7.3 (6.5, 8.1)	15.1 (14.0, 16.4)	19.4 (18.0, 20.9)
Regular alcohol use	8.2 (7.4, 9.1)	7.4 (6.6, 8.3)	21.4 (20.0, 22.8)	17.8 (16.5, 19.1)
Ever tried cannabis	10.4 (9.5, 11.4)	8.9 (8.1, 9.8)	29.6 (28.2, 31.2)	26.5 (25.1, 28.0)
Delinquency	34.2 (32.7, 35.7)	23.2 (21.9, 24.5)	27.3 (25.8, 28.8)	16.4 (15.3, 17.6)
Drinking and Smoking	1.6 (1.3, 2.0)	2.0 (1.6, 2.4)	7.3 (6.5, 8.2)	8.6 (7.7, 9.6)
Reg. alcohol use and tried cannabis	2.9 (2.4, 3.4)	2.8 (2.3, 3.4)	13.0 (11.9, 14.2)	11.4 (10.4, 12.6)
Smoking and tried cannabis	2.8 (2.4, 3.3)	4.0 (3.5, 4.6)	11.4 (10.4, 12.5)	13.6 (12.6, 14.8)
Delinquency and reg. alcohol use	5.1 (4.6, 5.8)	4.6 (4.0, 5.2)	11.2 (10.2, 12.3)	7.7 (6.8, 8.6)
Delinquency and smoking	3.8 (3.2, 4.5)	5.2 (4.6, 6.0)	9.3 (8.4, 10.4)	8.0 (7.1, 8.9)
Delinquency and drugs	7.8 (7.0, 8.6)	6.0 (5.4, 6.8)	15.8 (14.6, 17.1)	10.3 (9.3, 11.4)
Two or more risk behaviours	11.5 (10.6, 12.6)	11.0 (10.1, 11.9)	25.6 (24.2, 27.1)	21.9 (20.5, 23.4)

**Table 2:** Prevalence of risk behaviours by gender at age 19

	Age 19	
	Male	Female
	*N* = 4,684	*N* = 4,864
	% (95 % CI)	% (95 % CI)
Heavy alcohol use	24.6 (22.9, 26.4)	16.7 (15.3, 18.2)
Drug use in last month	18.5 (17.1, 19.9)	9.6 (8.6, 10.8)
Sexual risk	15.6 (14.3, 17.0)	11.6 (10.5, 12.9)
Heavy alcohol use and Drugs	7.5 (6.6, 8.5)	4.0 (3.3, 4.9)
Sex and heavy alcohol use	5.2 (4.5, 6.1)	2.8 (2.3, 3.5)
Sex and drugs	5.7 (4.9, 6.6)	2.0 (1.6, 2.6)
Two or more risk behaviours	13.4 (12.3, 14.7)	6.9 (6.0, 7.8)

At all time-points, individual and concurrent risk behaviours were higher in males except for smoking and most combinations involving smoking (the exception being concurrent delinquency and smoking at age 16), which were higher for females. The prevalence of all individual risk behaviours rose between age 14 and 16 except for delinquency, the prevalence of which decreased. (Those at age 19 were not directly comparable because they were defined differently.)

### Association between health risk behaviours

The associations between each risk behaviour at each reporting period are shown in Tables 3. In all cases, involvement in any risk behaviour was significantly associated with all other risk behaviours. This is reflected in the fact that prevalences of any risk behaviour for those involved in any other risk behaviour were many times higher than those not involved in that risk behaviour. The strength of association between risk behaviours was equal or greater at age 14 than at 16 for all associations excepting delinquency and alcohol use. Significant reductions in the strength of associations (based on non-overlapping odds ratios) were found for regular smoking with having ever tried cannabis, and regular smoking with delinquency. Though associations are not directly comparable between age 19 and earlier reporting periods because risk behaviours were defined differently, the odds-ratios at age 19 were uniformly smaller than in earlier waves. The highest associations at age 14 and 16 were found between cannabis and smoking. The strength of association between smoking and delinquency at age 14 was also particularly strong. At age 19, the association between sexual risk behaviour and heavy alcohol use was smaller than for other associations. For other cases, the sizes of odds ratios within each wave were largely comparable.

**Table 3:** Associations among risk behaviours at ages 14, 16 and 19

	Engagement in other risk behaviours			
	Reg. smoking	Reg. alcohol use	Ever tried cannabis	Delinquency
	% (95 % CI)	% (95 % CI)	% (95 % CI)	% (95 % CI)
Panel A: Age 14				
Reg. smokers	–	30.0 (26.3, 34.0)	58.3 (54.0, 62.6)	77.2 (73.4, 80.6)
Non-regular smokers	–	6.4 (5.8, 7.0)	6.5 (6.0, 7.1)	25.5 (24.5, 26.5)
**Association: OR**	–	**6.0 (4.8, 7.5)**	**22.2 (18.0, 27.3)**	**11.0 (8.8, 13.7)**
Reg. alcohol users	22.9 (19.9, 26.2)	–	36.9 (33.3, 40.6)	62.3 (58.9, 65.5)
Not regular users	4.5 (4.0, 5.1)	–	7.5 (6.9, 8.1)	25.7 (24.6, 26.8)
**Association: OR**	**6.0 (4.8, 7.5)**	–	**6.8 (5.6, 8.1)**	**4.7 (4.0, 5.5)**
Tried Cannabis	35.6 (32.7, 38.7)	29.1 (26.3, 32.0)	–	71.4 (68.5, 74.1)
Not tried Cannabis	2.7 (2.3, 3.1)	5.4 (4.9, 5.9)	–	23.8 (22.8, 24.8)
**Association: OR**	**22.3 (18.1, 27.5)**	**6.8 (5.6, 8.1)**	–	**7.4 (6.3, 8.6)**
Delinquency	15.8 (14.3, 17.5)	17.0 (15.7, 18.5)	24.3 (22.7, 26.0)	–
No delinquency	1.9 (1.6, 2.2)	4.1 (3.4, 4.7)	3.9 (3.4, 4.4)	–
**Association: OR**	**11.1 (8.9, 13.8)**	**4.7 (4.0, 5.5)**	**7.4 (6.3, 8.6)**	–
Panel B: Age 16				
Reg. smokers	–	46.2 (43.3, 49.1)	73.0 (70.7, 75.3)	50.1 (47.3, 52.9)
Non-regular smokers	–	13.9 (12.9, 14.8)	18.2 (17.2, 19.3)	15.5 (14.6, 16.4)
**Association: OR**	–	**5.3 (4.7, 6.1)**	**13.0 (11.4, 14.9)**	**6.1 (5.3, 7.0)**
Reg. alcohol users	40.1 (38.3, 43.6)	–	62.7 (60.1, 65.2)	48.1 (45.4, 50.8)
Not regular users	11.5 (10.6, 12.4)	–	20.0 (19.0, 21.0)	15.6 (14.7, 16.5)
**Association: OR**	**5.3 (4.6, 6.1)**	–	**6.4 (5.6, 7.2)**	**5.2 (4.6, 5.9)**
Tried cannabis	45.4 (43.1, 47.7)	43.3 (41.1, 45.5)	–	46.4 (44.3, 48.6)
Not tried	6.4 (5.8, 7.1)	10.2 (9.4, 11.0)	–	12.2 (11.4, 13.0)
**Association: OR**	**13.1 (11.4, 14.9)**	**6.4 (5.7, 7.2)**	–	**6.4 (5.7, 7.2)**
Delinquency	40.3 (37.9, 42.7)	43.0 (40.6, 45.4)	60.0 (57.6, 62.2)	–
No delinquency	11.0 (10.1, 11.9)	13.0 (12.1, 14.0)	19.3 (18.3, 20.3)	–
**Association: OR**	**6.1 (5.3, 7.0)**	**5.2 (4.6, 5.9)**	**6.4 (5.7, 7.2)**	–
	Engagement in other risk behaviours			
	Heavy alcohol use	Drug use, 30 days	Sexual Risk	
	% (95 % CI)	% (95 % CI)	% (95 % CI)	
Panel C: Age 19				
Heavy alcohol use	–	28.2 (25.8, 30.7)	19.3 (17.1, 21.7)	
Non-heavy or no use	–	10.5 (9.6, 11.4)	12.1 (11.2, 13.2)	
**Association: OR**	–	**3.1 (1.7, 2.3)**	**1.9 (1.6, 2.2)**	
Drugs	40.7 (37.4, 44.1)	–	26.8 (23.8, 30.1)	
No drugs	17.0 (15.9, 18.3)	–	11.3 (10.4, 12.3)	
**Association: OR**	**3.1 (2.6, 3.6)**	–	**2.7 (2.3, 3.3)**	
Sexual Risk	29.3 (26.3, 32.6)	28.2 (25.2, 31.5)	–	
No sexual risk	19.3 (18.1, 20.6)	12.1 (11.2, 13.0)	–	
**Association: OR**	**1.9 (1.6, 2.2)**	**2.7 (2.3, 3.3)**	–	

Prevalence of risk behaviours (%) by other risk involvement and odds-ratios (ORs; in bold) for the association between risk behaviours, controlled for gender, ethnicity and area deprivation (English Index of Multiple Deprivation; IMD). Panel A shows associations at age 14, Panel B shows associations at age 16 and Panel C shows associations at age 19

Because increasing associations between risk behaviours with age may be partly attributable to higher rates of attrition for those participating in risk behaviours, we examined associations between risk behaviours at age 14 and 16 using a complete-case sample of participants who completed the Wave 6 assessment. Odds ratios (not shown) were highly comparable to those in the original analysis and patterns of decreasing associations between risk behaviours with age held.

### Predictors of age 19 risk behaviour

Odds Ratios and *p*-values for the association between age 19 risk behaviours and earlier risk behaviour, demographics and interpersonal, school and family factors are shown in Table 4. Males were significantly more likely to be involved in all risk behaviours. Low parental education predicted unsafe sex but was inversely associated with heavy alcohol use and unassociated with drug use. Similarly, compared to the least deprived IMD quintile all others were significantly associated with unsafe sex and all quintiles excepting the second least deprived were inversely associated with heavy alcohol use. IMD was unassociated with drug use. Compared to white ethnicity, Asian ethnicity was protective for all risk behaviours as were black and ‘other’ ethnicities for heavy alcohol use. Mixed ethnicity was protective for heavy alcohol use but was associated with increased drug use. Other comparisons failed to reach significance.

**Table 4:** Adjusted associations of early risk behaviour, sociodemographic and psychosocial factors with age 19 unsafe sex, heavy alcohol use and drug use

	Unsafe sex		Heavy alcohol use		Drug use	
	OR (CI)	*P*	OR (CI)	*P*	OR (CI)	*P*
Sociodemographics						
Gender (ref: Male)	.71 (.61, .81)	<.001	.61 (.53, .69)	<.001	.47 (.40, .54)	<.001
Low parental education	1.31 (1.08, 1.58)	.006	.52 (.43, .63)	<.001	.98 (.81, 1.20)	.862
IMD (ref: least deprived)						
2nd least deprived	1.28 (1.01, 1.61)	.04	.87 (.74, 1.01)	.067	1.06 (.88, 1.27)	.535
Median quintile	1.56 (1.23, 1.97)	<.001	.76 (.64, .90)	.002	.99 (.79, 1.23)	.923
2nd most deprived	2.27 (1.79, 2.87)	<.001	.62 (.51, .75)	<.001	1.07 (.85, 1.34)	.584
Most deprived	2.26 (1.74, 2.95)	<.001	.43 (.34, .55)	<.001	.98 (.76, 1.27)	.886
Ethnicity (ref: White)						
Asian	.16 (.11, .23)	<.001	.35 (.26, .46)	<.001	.32 (.24, .43)	<.001
Black	.57 (.40, .80)	.001	.25 (.17, .37)	<.001	.55 (.35, .88)	.012
Mixed	.89 (.62, 1.29)	.551	.80 (.56, 1.13)	.198	1.79 (1.31, 2.45)	<.001
Other	.45 (.24, .85)	.014	.43 (.34, .55)	<.001	.41 (.21, .78)	.007
Risk behaviours						
Regular alcohol use						
Age 14 only	1.95 (1.33, 2.87)	.001	1.45 (1.04, 2.04)	.031	1.70 (1.17, 2.49)	.006
Age 16 only	1.99 (1.60, 2.46)	<.001	2.24 (1.90, 2.65)	<.001	3.06 (2.52, 3.71)	<.001
Both age 14 and 16	3.00 (2.18, 4.13)	<.001	3.82 (2.93, 4.98)	<.001	3.96 (2.92, 5.35)	<.001
Regular smoking						
Age 14 only	2.68 (1.16, 6.15)	.021	1.02 (.45, 2.32)	.955	2.54 (1.12, 5.75)	.025
Age 16 only	2.99 (2.43, 3.70)	<.001	1.49 (1.24, 1.81)	<.001	4.44 (3.65, 5.41)	<.001
Both age 14 and 16	3.25 (2.32, 4.55)	<.001	1.06 (.73, 1.54)	.753	5.28 (3.87, 7.19)	<.001
Delinquency						
Age 14 only	2.20 (1.76, 2.75)	<.001	1.03 (.85, 1.25)	.757	2.08 (1.69, 2.54)	<.001
Age 16 only	2.54 (1.98, 3.24)	<.001	1.40 (1.12, 1.74)	.003	2.58 (2.06, 3.22)	<.001
Both age 14 and 16	4.33 (3.49, 5.36)	<.001	1.65 (1.34, 2.02)	<.001	5.32 (4.34, 6.53)	<.001
Tried cannabis						
Tried by age 14	2.71 (2.16, 3.41)	<.001	1.80 (1.47, 2.22)	<.001	7.81 (6.25, 9.75)	<.001
Tried by age 16	2.22 (1.84, 2.67)	<.001	2.13 (1.83, 2.47)	<.001	5.29 (4.47, 6.26)	<.001
Age 14 risk score						
One	2.13 (1.76, 2.56)	<.001	1.34 (1.15, 1.55)	<.001	2.42 (2.03, 2.88)	<.001
Two	2.90 (2.21, 3.81)	<.001	1.49 (1.15, 1.94)	.002	4.15 (3.19, 5.38)	<.001
Three	4.12 (2.87, 5.90)	<.001	1.32 (.93, 1.88)	.118	6.83 (4.96, 9.40)	<.001
Four	4.36 (2.22, 8.58)	<.001	2.84 (1.51, 5.37)	.001	8.73 (4.59, 16.60)	<.001
Age 16 risk score						
One	1.93 (1.58, 2.36)	<.001	1.81 (1.55, 2.10)	<.001	3.23 (2.65, 3.93)	<.001
Two	3.14 (2.48, 3.96)	<.001	2.30 (1.91, 2.78)	<.001	5.98 (4.84, 7.39)	<.001
Three	3.75 (2.93, 4.80)	<.001	2.53 (2.00, 3.21)	<.001	10.70 (8.38, 13.66)	<.001
Four	6.05 (4.36, 8.38)	<.001	2.73 (2.04, 3.67)	<.001	14.10 (10.47,18.99	<.001
Interpersonal factors						
GHQ (age 15)	1.52 (1.26, 1.83)	<.001	1.28 (1.10, 1.50)	.002	1.72 (1.43, 2.06)	<.001
Friends over often (age 14)	1.42 (1.20, 1.68)	<.001	.93 (.81, 1.06)	.271	1.37 (1.16, 1.60)	<.001
Out with friends often (age 14)	1.79 (1.54, 2.08)	<.001	1.01 (.89, 1.15)	.856	1.53 (1.33, 1.75)	<.001
Bullied in last 12 months (age 14)	1.28 (1.09, 1.50)	.002	1.02 (.90, 1.16)	.740	1.34 (1.15, 1.55)	<.001
School factors						
Low academic attainment (age 14)	1.71 (1.43, 2.05)	<.001	.62 (.51, .74)	<.001	.95 (.77, 1.16)	.590
Self-reported bad at school (age 14)	1.92 (1.64, 2.24)	<.001	.82 (.72, .93)	.002	1.29 (1.10, 1.50)	.001
Exclusions in past 3 years (age 14)	1.95 (1.52, 2.52)	<.001	1.03 (.80, 1.32)	.839	2.08 (1.63, 2.64)	<.001
Truancy in last 12 months (age 14)	2.11 (1.72, 2.60)	<.001	1.31 (1.10, 1.57)	.003	2.80 (2.34, 3.36)	<.001
Attitudes towards school (age 14; continuous)	.95 (.94, .96)	<.001	.99 (.98, 1.00)	.011	.96 (.95, .97)	<.001
Family factors						
Single parent family (age 14)	1.37 (1.15, 1.64)	.001	.87 (.73, 1.03)	.113	1.31 (1.10, 1.57)	.003
Not get on very well with parents (age 14)	1.22 (1.03, 1.44)	.022	1.12 (.98, 1.29)	.105	1.21 (1.03, 1.41)	.020
Low parental communication (age 14)	1.31 (1.11, 1.54)	.001	1.11 (.97, 1.27)	.129	1.41 (1.21, 1.63)	<.001
Parents not always know where child is in evenings (age 14)	1.81 (1.55, 2.10)	<.001	1.24 (1.09, 1.40)	.001	1.93 (1.68, 2.21)	<.001

All analyses were adjusted for ethnicity, parental education, area deprivation (English Index of Multiple Deprivation; IMD) and gender. Associations for all other items are modeled separately

*Ref* reference group. Where not stated, the reference group for all other categorical variables is the absence of the stated factor. All predictor variables are binary except “attitudes towards school”

All age 14 and 16 risk behaviours were significantly associated with age 19 unsafe sex, drug use and heavy alcohol use except for age 14 only smoking which was not significantly associated with alcohol use, and age 14 only delinquency which was unassociated with heavy alcohol use. In all cases, increasing risk scores were increasingly associated with age 19 risk behaviours.

GHQ score was positively associated with all risk behaviours. Having friends over often, being out with friends often and being bullied predicted all risk behaviours excepting heavy alcohol use.

Good grades were associated with increased risk for alcohol use, but were protective for unsafe sex. Grades were unassociated with illicit drug use. Low self-rated scholastic ability was associated with increased risk for unsafe sex and drug use, but with decreased risk for heavy alcohol use. Truancy was associated with all risk behaviours at 19. School exclusions were associated with all risk behaviours excepting alcohol use. Positive attitudes towards school were associated with decreased risk for all risk behaviours.

Living in single-parent households was a significant risk factor for all risk behaviours excepting heavy alcohol use. The same patterns emerged for not getting along with parents and parental communication. Parents not always knowing where the respondent is in the evening was a risk factor for all risk behaviours.

### Comparing early risk behaviours as predictors for multiple risk behaviour

Two chi-square analyses were conducted with the aim of comparing early risk behaviours to ascertain which were most strongly associated with multiple risk behaviour at age 19. The results show that early risk behaviour was associated with multiple risk behaviour at 19 for both age 14 risk behaviour, *χ*^2^ (5) = 405.06, *p* < .001 and age 16 risk behaviour, *χ*^2^ (5) = 764.25, *p* < .001. We compared observed proportions of respondents involved in multiple risk behaviour based on age 14 and age 16 patterns of risk behaviour with expected proportions of multiple risk behaviour. (Expected proportions were calculated as the overall total rate of multiple risk behaviour involvement: 10.1 %.) Those not involved in any risk behaviour at age 14 or 16 were underrepresented in age 19 multiple risk behaviour, while those involved in more than one risk behaviour were overrepresented (Table 5). Those who only smoke at age 14 were slightly underrepresented, while those who smoke only at age 16 were substantially less likely to engage in multiple risk behaviour than average. Those who drank only at 14 were most likely than average to become multiple risk takers at 14 but those who drank only at 16 were less likely than average. A similar pattern emerged for delinquency, though observed and expected percentages of multiple risk behaviour were near identical for age 16 delinquency. Having tried cannabis was the only individual risk behaviour for which observed percentages for age 19 multiple risk behaviour were higher than expected at both age 14 and 16.

**Table 5:** Expected and observed percentage of respondents involved in age 19 multiple risk behaviour (2 or more behaviours) by age 14 and age 16 risk behaviour

	Expected (%)	Age 14		Age 16	
		Observed (%)	AR	Observed (%)	AR
No risk behaviours	10.1	6.1	−16.68	3.8	−19.97
Regular smoking	10.1	8.7	−0.35	5.8	−2.40
Regular drinking	10.1	15.3	2.34	7.7	−1.63
Tried Cannabis	10.1	22.4	5.32	16.0	4.15
Delinquency	10.1	14.6	6.08	9.5	−0.29
Multiple risk (2+)	10.1	24.4	14.44	24.8	23.04

*AR* adjusted residuals, indication of magnitude of difference between expected and observed counts. ARs over 2 and below −2 indicate statistically significant differences between expected and observed proportions. Positive ARs indicate larger observed proportions than expected; negative ARs indicate smaller observed proportions than expected

## Discussion

These data from a large nationally representative contemporary dataset demonstrate strong associations between health risk behaviours in adolescents, though the strength of associations decreased with age. The strongest associations tended to be with drug use and the weakest with alcohol use as hypothesised. Risk factors for drug use and unsafe sex were comparable; patterns of risk factors for heavy alcohol use often differed. This corresponds with our proposed hypotheses regarding perceptions of alcohol use as socially normative. Those involved in multiple risk behaviour in early adolescence were far more likely to be multiple risk-takers at age 19, with single-risk takers in mid-adolescence largely underrepresented in age 19 multiple risk behaviour.

Our findings fit with gateway theories by suggesting that few drug users are not also involved in other “stepping stone” behaviours, such as tobacco and alcohol use [19] and that drinking and smoking in early adolescence were strongly predictive of drug use in late adolescence. The findings are also interpretable from the perspective of problem behaviour theory, whereby different forms of adolescent risk behaviour are manifestations of unconventional behaviour; this is consistent with alcohol having weaker associations with other risk behaviours because, arguably, it is among the most socially accepted form of risk behaviour. Further, though early alcohol use predicted other forms of later risk behaviour strongly, other risk behaviours in early adolescence were relatively weak predictors of age 19 drinking. Since respondents were of legal drinking age at this age, social disapproval for drinking may be perceived as particularly low. Other included risk behaviours, such as adolescent smoking, delinquency and drug use, and adult unsafe sex and drug use are generally socially (or legally) proscribed. The pattern of associations supports multiple risk behaviour as a rejection of social norms.

Despite strong associations between risk behaviours at all reporting periods, the size of associations decreased with age. Past studies show that associations between risk behaviours are substantially lower in late adolescence compared to earlier stages of adolescence [16, 33]. This may represent a transition from general risk behaviour etiology to risk-specific influences [25]. At-risk adolescents appear to participate in risk behaviour indiscriminately. From a problem behaviour perspective, risk behaviour in adolescence co-occurs because it serves comparable functions, relating to sensation-seeking, defying convention and demonstrating maturity and independence. This may be related to higher rates of impulsivity, and risk-taking compared to adults. Deficits in impulse-control have a neurological basis due to ongoing maturation in the prefrontal cortex [34].

The weakening association between risks seems at odds with gateway theories which might suggest that associations between risks become stronger as early risk behaviours led to opportunities to engage in others. Relatedly, multiple risk behaviour initiation seems to occur early; age 14 and age 16 multiple risk takers were far more likely than others to be involved in multiple risk behaviour in late adolescence. Past work has noted that those who initiate risk behaviours early are more likely to be multiple risk-takers [35].

Along with previous health risk behaviours, we identified a number of sociodemographic, interpersonal, school and family risk and protective factors for risk behaviours in late adolescence. These generally correspond with findings of previous studies [13, 14, 35–37]. This gives credence to the notion that multiple risk behaviours arise due to common risk and protective factors. The identified common risk factors complement and contribute to existing evidence regarding common risk factors for substance use, delinquency and unsafe sexual practices [11, 14, 17, 38, 39].

Some key differences are evident when comparing alcohol use to other risk behaviours. Our findings correspond with past studies which have noted that alcohol use (but not other risk behaviours) is associated with increased affluence [40, 41]. This may also be related to the differences found in regards to school factors. Past research has found that heavy alcohol use in early adolescence is related to poor academic performance, but in late adolescence the association disappears or is reversed [42]. Heavy alcohol use in late adolescence is associated with different alcohol use trajectories. Some represent pervasive and continuing heavy use, while others reflect the contextual and social norms of late adolescence [43]. These trajectories are associated with different socio-demographic characteristics and educational outcomes. Those in postsecondary education are often involved in more heavy drinking than others, despite less alcohol use in early adolescence [43].

Our results suggest that common risk factors are associated with a range of risk behaviours. This suggests that interventions targeting risk factors for individual risk behaviours may also have implications for other (non-targeted) risk behaviours and multiple risk behaviours. The findings also have implications for the timing of such interventions, since multiple risk behaviour is often initiated early in adolescence. However, a large proportion of risk-takers are involved in only one type of risk meaning that risk-specific interventions are indispensible. Most risk behaviour fits a pattern of non-conventional behaviour manifesting in unhealthy behaviour. However, alcohol use in late adolescence appears to defy this pattern in some regards necessitating strategies which reflect its different range of risk and protective factors. As such, our findings are highly consistent with explanations of adolescent risk behaviour based on problem behaviour theory.

### Limitations

Our study was strengthened by the use of a large, demographically representative national dataset containing longitudinal data throughout adolescence. These data focused on a limited selection of risk behaviours. The definition of multiple-risk behaviour was based on this limited selection and as such, differs from some past definitions [44–46]. Though the findings are likely generally applicable across a wider range of risk factors, this requires replication using a broader range of risk behaviours. Risk behaviours available in earlier waves were not available at age 19 so we could not assess the prevalence of multiple risk behaviour at age 19 in a comparable way to earlier waves.

Defining multiple risk behaviour as involvement in any two risk behaviours also presents some problems because it treats all combinations of risk behaviours as equivalent. Likewise, this weakness applies to the construction of risk scores at age 14 and 16, though sensitivity analyses suggest that excluding each individual risk behaviour from the computation of the risk score leads to similar associations with age 19 risk behaviours. Measuring risk behaviour participation entirely based on self-reports may threaten the validity of the findings. However, studies comparing self-report to objective measures of risk behaviour in adolescents suggests that self-report measures provide largely valid data [47, 48]. Though a number of domains of risk factors for health risk behaviours were represented within the data set, several important predictors were unavailable such as impulsivity, sensation-seeking and peer risk behaviours.

The analyses involved a relatively large number of associations among pairs of variables and are thus liable to Type I errors. This limitation is somewhat tempered by the strong effects noted for the majority of significant associations, with *P*-values frequently less than .001.

Attrition may have compromised the validity of the findings. Total attrition between waves 1 and 6 was nearly 40 %. This may have changed the pattern of results, especially considering the differences in attrition across socio-demographic variables and participation in risk behaviour. We used sample weights to partially attenuate this limitation. These weights accounted for differences in response rates based on risk behaviours including substance use and sexual risk behaviour. Furthermore, we repeated analyses regarding the strength of associations across adolescence (which we identified as particularly vulnerable to bias due to attrition) with a complete-case sample with identical results, suggesting that changes across adolescence in the strength of associations among risk behaviours are not due to attrition. As such, we believe the bias introduced by attrition to be minimal.

## Conclusion

Adolescent risk behaviours are strongly linked, though the strength of association decreases with age. This link is at least partially attributable to common risk and protective factors, though late adolescent alcohol use does not conform to the patterns established by other risk behaviours. Multiple risk behaviours are initiated early in adolescence with some adolescents initiating a single risk behaviour in early or mid adolescence and not progressing to others. The findings are suggestive of interventions targeting risk-taking as a manifestation of non-conventional behaviour which, in conjunction with single-risk behaviour prevention strategies, have the potential to reduce multiple risk behaviour by targeting common risk factors.

## Abbreviations

GHQ, General Health Questionnaire; IMD, Index of Multiple Deprivation; LSYPE, Longitudinal Study of Young People in England; NPD, National Pupil Data.
